# Value of pyrazinamide for composition of new treatment regimens for multidrug-resistant *Mycobacterium tuberculosis* in China

**DOI:** 10.1186/s12879-020-4758-9

**Published:** 2020-01-07

**Authors:** Hui Xia, Susan van den Hof, Frank Cobelens, Yang Zhou, Bing Zhao, Shengfen Wang, Yanlin Zhao

**Affiliations:** 1 National Tuberculosis Reference Laboratory, National Center for Tuberculosis Control and Prevention, Chinese Center for Disease Control and Prevention, Beijing, China; 2 KNCV Tuberculosis Foundation, The Hague, The Netherlands; 3 National Institute of Public Health and the Environment, Centre for Infectious Disease Epidemiology and Surveillance, Bilthoven, The Netherlands; 4 Department of Global Health and Amsterdam Institute for Global Health and Development, Amsterdam University Medical Centers, Amsterdam, Netherlands

**Keywords:** Multidrug-resistant tuberculosis, Pyrazinamide, Resistance, *pncA*

## Abstract

**Background:**

Pyrazinamide still may be a useful drug for treatment of rifampin-resistant (RR-TB) or multidrug-resistant tuberculosis (MDR-TB) in China while awaiting scale up of new drugs and regimens including bedaquiline and linezolid. The level of pyrazinamide resistance among MDR-TB patients in China is not well established. Therefore, we assessed pyrazinamide resistance in a representative sample and explored determinants and patterns of *pnc*A mutations.

**Methods:**

MDR-TB isolates from the 2007 national drug resistance survey of China were sub-cultured and examined for pyrazinamide susceptibility by BACTEC MGIT 960 method. *pnc*A mutations were identified by sequencing. Characteristics associated with pyrazinamide resistance were analyzed using univariable and multivariable log-binominal regression.

**Results:**

Of 401 MDR-TB isolates, 324 were successfully sub-cultured and underwent drug susceptibility testing. Pyrazinamide resistance was prevalent in 40.7% of samples, similarly among new and previously treated MDR-TB patients. Pyrazinamide resistance in MDR-TB patients was associated with lower age (adjusted OR 0.54; 95% CI, 0.34–0.87 for those aged ≧60 years compared to < 40 years). Pyrazinamide resistance was not associated with gender, residential area, previous treatment history and Beijing genotype. Of 132 patients with pyrazinamide resistant MDR-TB, 97 (73.5%) had a mutation in the *pncA* gene; with 61 different point mutations causing amino acid change, and 11 frameshifts in the *pncA* gene. The mutations were scattered throughout the whole *pnc*A gene and no hot spot region was identified.

**Conclusions:**

Pyrazinamide resistance among MDR-TB patients in China is common, although less so in elderly patients. Therefore, pyrazinamide should only be used for treatment of RR/MDR-TB in China if susceptibility is confirmed. Molecular testing for detection of pyrazinamide resistance only based on *pncA* mutations has certain value for the rapid detection of pyrazinamide resistance in MDR-TB strains but other gene mutations conferring to pyrazinamide resistance still need to be explored to increase its predictive ability .

## Background

Multidrug-resistant tuberculosis (MDR-TB) including extensively drug-resistant tuberculosis (XDR-TB) has emerged as one of the health threats in China. The proportion of new and previously treated TB patients with MDR was 5.7 and 25.6%, respectively [1]. Pyrazinamide, a first-line drug with bactericidal activity, has an important role in the treatment of both drug-susceptible and drug-resistant tuberculosis. Although its role in the longer MDR-TB regimen has been limited with the availability of new core drugs, such as bedaquiline and linezolid, its role in novel shorter regimens currently under trial but with promising outlooks, is pivotal. Anti-tuberculosis regimens consisting of moxifloxacin, pyrazinamide and other drugs for shortening treatment of multi-drug resistant tuberculosis were evaluated and used [2–5] before the new guideline was issued by WHO. The new WHO guidelines (2018) recommend that fully oral longer regimens would include at least four agents likely to be effective in the first 6 months and three thereafter. All three group A agents including fluoroquinolones (levofloxacin or moxifloxacin), bedaquiline and linezolid and at least one group B agent should be included. If the regimen cannot be composed with agents from Groups A and B alone, the regimens are completed with Group C agent [6]. Bedaquiline is not widely used in China and only offered in very few provincial and prefecture level hospitals. Linezolid has a very high market price in China, which may bring some obstacles to nationwide use. Therefore, in China group C agents including pyrazinamide may still be used to compose both longer and shorter regimen for drug resistant TB treatment.

Pyrazinamide is a prodrug that requires conversion into pyrazinoic acid by the bacterial enzyme pyrazinamidase/nicotinamidase encoded by *pncA gene in M.tuberculosis* [7]. Mutations in *pncA* encoding pyrazinamidase lead to the loss of pyrazinamidase activity and are the major mechanism of pyrazinamide resistance in *M. tuberculosis* [7, 8]. Pyrazinamide has no activity in vitro at neutral pH but is active at an acid pH (pH 5.5) [9,10]. Pyrazinamide susceptibility testing is difficult because the acidity of culture medium needed for drug activity also restricts the growth of *M. tuberculosis*. Because of these technical challenges, drug susceptibility for pyrazinamide is seldomly performed in routine settings in many countries including China. This partly explains the paucity of data on the prevalence of pyrazinamide resistance among MDR-TB patients in China. The available data show a large variation in pyrazinamide prevalence, which may be explained through inclusion of selected cases [11–14]. In this study, we analyzed pyrazinamide resistance, risk factors for resistance, and mutations in the *pncA* gene, among patients identified with MDR-TB in a nationally representative sample of TB patients in China.

## Methods

### Patients and mycobacterial isolates

A nationally representative sample of 3634 smear-positive pulmonary tuberculosis patients were included in the 2007 national drug resistance survey, of which 401 (11.0%) cases were identified as MDR-TB cases with the proportion method on Löwenstein-Jensen medium [1]. Out of the 401 MDR-TB strains, 75 strains failed to grow in subculture and 2 strains failed on pyrazinamide susceptibility testing, leaving 324 MDR-TB strains for analysis. These 324 patients were from 30 of all 31 provinces in China (except for Hainan province). The mean age (standard deviation) of the 324 MDR-TB patients included for final analysis was 43.0 (SD 16.5) years and 211 (65.1%) were male. Ethics approval of this study was waived by the Ethical Committees of the Chinese Center for Disease Control and Prevention. The 2007 national drug resistance survey was approved by the Tuberculosis Research Ethics Review Committee of the Chinese Center for Disease Control and Prevention and written informed consent was obtained from each participant.

### Patient information

Basic demographic data including age, gender, occupation, area of residence within China, residence in an area with DOTS implementation before 2000 or afterwards, previous anti-TB treatment episodes and previous pyrazinamide usage were collected from the standardized questionnaire used in survey [1].

### Drug susceptibility testing

Susceptibility to pyrazinamide was determined with the BACTEC MGIT 960 system (Becton Dickinson) as recommended by the manufacturer. The medium was modified Middlebrook 7H9 broth (pH 5.9) containing 100 μg/ml pyrazinamide. *Mycobacterium bovis* BCG (ATCC 34540) and *Mycobacterium tuberculosis* H37Rv (ATCC 27294) were used as pyrazinamide resistant and susceptible controls, respectively. Susceptibility to ofloxacin and kanamycin had been tested with the proportion method on L-J medium in 2007 [1].

### Amplification and sequencing of the *pncA* gene

DNA Sanger sequencing of the *pncA* gene and its upstream region was performed on all isolates. DNA was extracted from isolates by transferring one loopful of bacteria from L-J slants to a 1.5-ml tube containing 1 ml of Tris-EDTA buffer. Each tube was vortexed for 30 s and placed in a heat block at 80 °C for at least 30 min for inactivation. Tubes were then moved to 100 °C for 15 min and then centrifuged. The supernatant was kept for *pncA* amplication. The entire pncA gene and 82 bp of an upstream putative regulatory region were amplified as described previously [8]. The primers were as follows: PNCA-F: GTC GGT CAT GTT CGC GAT CG, PNCA-R: GCT TTG CGG CGA GCG CTC CA. The PCR cycling parameters were 95 °C for 5 min, followed by 30 cycles of 95 °C for 1 min, 55 °C for 1 min, and 72 °C for 1 min. PCR was performed in a total volume of 25 μl, and the PCR reaction mixture consisted of 12.5ul 2 × Taq Master Mix (Kangwei, China), 20 pmol of each primer and 2 μl of DNA. Finally, PCR products were directly sequenced using the PNCA-F primers as sequencing primers. The sequencing results were entered into the Basic Local Alignment Search Tool (BLAST, https://blast.ncbi.nlm.nih.gov/Blast.cgi) for comparison with the *pncA* gene of the *Mycobacterium tuberculosis* H37Rv.

Synonymous single-nucleotide polymorphisms (sSNP), or nonsynonymous single-nucleotide polymorphisms (nSNP) in the sequencing results of *pncA* gene that had been reported not to be related to pyrazinamide resistance [15] were excluded when calculating specificity of the genetic mutation. Strains with all other SNPs or insertions or deletions were considered mutant and likely were resistant to pyrazinamide.

### Genotyping

Beijing or non-beijing strains were distinguished based on spoligotyping, as described previously [16]. The original binary data were submitted to the SITVITWEB database to obtain the spoligotype.

### Statistical analysis

Patients with pyrazinamid resistant *M. tuberculosis* strains were compared with patients with pyrazinamide susceptible strains with regard to the characteristics provided in the questionnaire used in 2007 drug resistance survey [1] and *M. tuberculosis* genotype, separately for phenotypically and genotypically determined resistance profiles. Univariable log-binominal regression modeling was used to calculate prevalence ratios (PRs) for factors associated with pyrazinamide resistance. Age, gender, and number of previous TB treatment episodes were fixed in the multivariable model and other characteristics with a *p*-value < 0.1 or plausibly (epidemiologically or biologically) associated with pyrazinamide resistance were included in the initial multivariable log-binominal regression model. The final model was determined using backward selection, guided by statistical significance (*p*-value < 0.05) of variables and fit of the model. Data was analyzed using IBM SPSS software, version 19.0.

## Results

### Phenotypic pyrazinamide resistance

Of 324 MDR-TB isolates, 132 (40.7%) were phenotypically resistant to pyrazinamide, coming from 25 provinces.

### Risk factors for phenotypic pyrazinamide resistance

Univariable analysis showed that MDR-TB cases with pyrazinamide resistance were more likely to be younger (crude PR for age ≥ 60 compared to < 40 years: 0.55; 95% CI, 0.35–0.86) and more likely to have non-farming occupations (crude PR: 1.34; 95% CI, 1.03–1.73). In multivariable analysis, only younger age was associated with pyrazinamide resistance (adjusted PR for age ≥ 60 compared to < 40 years: 0.54; 95% CI, 0.34–0.87) (Table 1). The database for Univariable and multivariable analysis of risk factors associated with phenotypic pyrazinamide resistance was available in Additional file 1.

**Table 1:** Univariable and multivariable analysis of risk factors for phenotypic pyrazinamide resistance

Characteristic	All cases n (%)	Pyrazinamide- susceptible n (%)	Pyrazinamide resistant n (%)	Crude Prevalence ratio (95% CI)	Adjusted Prevalence ratio (95%CI)	*P* value
Gender						
Male	211 (65.1)	129 (62.7)	82 (62.1)	1.0	1.0	
Female	113 (34.9)	63 (32.8)	50 (37.9)	1.14 (0.88–1.49)	1.08 (0.82–1.42)	0.59
Age (years)						
<40	153 (47.2)	82 (42.7)	71 (53.8)	1.0	1.0	
40–59	108 (33.3)	63 (32.8)	45 (34.1)	0.90 (0.68–1.19)	0.86 (0.65–1.14)	0.3
≧60	63 (19.4)	47 (24.5)	16 (12.1)	0.55 (0.35–0.86)	0.54 (0.34–0.87)	0.01
Residential area within China						
East	104 (32.1)	65 (33.9)	39 (29.5)	1.0	NA	
Central	162 (50.0)	94 (49.0)	68 (51.5)	1.12 (0.82–1.52)	NA	
West	58 (17.9)	33 (17.2)	25 (18.9)	1.45 (0.78–1.69)	NA	
Occupation						
Farmer	202 (62.3)	129 (67.2)	73 (55.3)	1.0	NA	
Other	122 (37.7)	63 (32.8)	59 (44.7)	1.34 (1.03–1.73)	NA	
Residence in area according to start DOTS implementation						
In 2000 or later	187 (57.7)	106 (55.2)	81 (61.4)	1.0	NA	
Before 2000	137 (42.3)	86 (44.8)	51 (38.6)	0.86 (0.66–1.13)	NA	
Number of previous anti-TB treatments						
0	132 (40.7)	79 (41.1)	53 (40.2)	1.0	1.0	
1	107 (33.0)	67 (34.9)	40 (30.3)	0.93 (0.68–1.28)	0.96 (0.70–1.33)	0.82
> 1	81 (25.0)	44 (22.9)	37 (28.0)	1.14 (0.83–1.56)	1.20 (0.88–1.64)	0.24
Unknown	4 (1.2)	2 (1.0)	2 (1.5)	1.25 (0.46–3.39)	1.30 (0.46–3.66)	0.62
Previous pyrazinamide usage						
Yes	144 (44.4)	87 (45.3)	57 (43.2)	1.0	NA	
No	180 (55.6)	105 (54.7)	75 (56.8)	1.06 (0.81–1.38)	NA	
Genotype						
Non-beijing strain	42 (13.0)	27 (14.1)	15 (11.4)	1.0	NA	
Beijing strain	230 (71.0)	133 (69.3)	97 (73.5)	0.90 (0.70–1.16)	NA	
Unknown	52 (16.0)	32 (16.7)	20 (15.2)	0.96 (0.70–1.31)	NA	

*NA* not applicable, these variables were not included in the final model

### Mutations in the *pncA* gene

Among 132 strains phenotypically resistant to pyrazinamide, nucleotide changes that include non-synonymous mutations, insertions and deletions in the *pncA* gene were detected in 97 (73.5%) isolates compared to 13 of 192 (6.8%) pyrazinamide susceptible isolates. The concordance between phenotypic and genotypic results was 85.3% (276/324). Only four point mutations (Met1Thr, Asp12Asn, His137Arg, Thr47Ala) were detected both in phenotypically resistant and in phenotypically susceptible strains. Among the 97 isolates with gene mutations or frameshifts, we found 61 different point mutations causing amino acid change, and 11 frameshift changes. In phenotypically susceptible isolates 15 non-synonymous mutations, 1 frameshift mutation was detected. The mutations were scattered throughout the whole *pnc*A gene and no hot spot regions were found. The frequency of mutations in different regions along the *pncA* gene is shown in Table 2.

**Table 2:** Characteristics of *pncA* gene mutations in MDR-TB patient isolates

Serial number	Amino acids changes	Phenotypic pyrazinamide susceptibility testing results		
		susceptible	resistant	total
1	A(−11)G	0	3	3
2	Met1Thr	2	2	4
3	Leu4Trp	0	1	1
4	Ile5Ser^b^	0	1	1
5	Val9Ala^b^	0	1	1
6	Gln10Pro	0	4	4
7	Gln10stop	1	0	1
8	Asp12Ala	0	1	1
9	Asp12Asn	2	1	3
10	Asp12Glu	0	1	1
11	His137Arg^b^	1	1	2
12	Cys14Gly^a^	1	0	1
13	Cys14Tyr^b^	0	1	1
14	Gly17Asp	0	1	1
15	Gly17Ser^b^	0	1	1
16	Leu27Pro	0	2	2
17	Tyr34Ser	0	1	1
18	Val44Gly	0	1	1
19	Thr160Pro	0	1	1
20	Thr47Ala^a^	1	1	2
21	Lys48Arg	1	0	1
22	Lys48Glu	0	3	3
23	Pro54Leu	0	2	2
24	His57Gln^b^	0	2	2
25	Pro62Leu	0	1	1
26	Pro62Pro(synonymous)^a^	1	0	1
27	Asp63Tyr/Thr142Lys^b^	0	1	1
28	Ser164Pro^b^	0	1	1
29	Ser65Ser(synonymous)^a^	1	0	1
30	Trp68Gly	0	1	1
31	Pro69Leu	1	0	1
32	His71Pro	0	1	1
33	Thr76Pro	0	3	3
34	Leu85Pro	0	2	2
35	Phe94Ser	0	2	2
36	Lys96Arg	0	1	1
37	Lys96Met^b^	0	1	1
38	Lys96Thr	0	1	1
39	Gly97Ser	0	1	1
40	Ser66Leu	1	0	1
41	Ala102Pro	0	1	1
42	Tyr103Cys	2	0	2
43	Tyr103Ser	1	0	1
44	Leu116Arg	0	1	1
45	Trp119Arg^b^	0	1	1
46	Gln122stop^b^	0	1	1
47	Val125Gly	0	1	1
48	Val130Ala^a^	1	0	1
49	Val130Met	1	0	1
50	Val139Gly	0	1	1
51	Gly132Asp	0	3	3
52	Ile133Thr	0	1	1
53	Asp136Asn	0	1	1
54	Asp136Gly	0	1	1
55	Cys138Trp^b^	0	2	2
56	Val139Leu	0	2	2
57	Arg140Gly^b^	0	1	1
58	Gln141Pro	0	2	2
59	Thr100Pro^b^	0	2	2
60	Thr142Ala	0	2	2
61	Ala146Thr^b^	0	2	2
62	Asn149Val	0	1	1
63	Arg154Trp^b^	0	1	1
64	Val155Met	1	0	1
65	Val157Gly^b^	0	1	1
66	Thr142Met	0	1	1
67	Ala161Gly^b^	0	1	1
68	Gly162Val	0	1	1
69	Ala171Val^a^	2	0	2
70	Leu172Pro^b^	0	1	1
71	Glu173Glu(synonymous)^a^	1	0	1
72	Glu174Gly^b^	0	1	1
73	Met175Ile	0	2	2
74	Met175Val	0	2	2
75	Leu182Ser^b^	0	1	1
76	Frameshift^b^	1	11	12
–	Wild type	169	35	204
–	Total	192	132	324

Note: ^a^ This synonymous single-nucleotide polymorphism (sSNP), or nonsynonymous single-nucleotide polymorphism (nSNP) not relating to pyrazinamide resistance (18) was excluded for calculating the concordance between genotypic mutations and phenotypic susceptible results

^b^ These mutations and all frameshift changes (insertions or deletions) relating to pyrazinamide resistance were first found in this study

### Risk factors for genotypic pyrazinamide resistance

Univariable analysis showed that younger age and Beijing genotype were associated with *pncA* gene mutations. In multivariable analysis, younger age and Beijing genotype were still risk factors for genotypic resistance (*pncA* mutation). The adjusted PR for patients aged≧60 compared to < 40 years was 0.56 (95% CI, 0.34–0.91) and for Beijing genotype was 1.84 (95% CI, 1.01–3.34) in comparison with non-Beijing genotype (Table 3). The database for Univariable and multivariable analysis of risk factors with genotypic pyrazinamide resistance was available in Additional file 1.

**Table 3:** Univariable and multivariable analysis of risk factors for genotypic pyrazinamide resistance (*pncA* mutation)

Characteristic	All cases n (%)	Wild type in *pncA* gene n (%)	Mutation in *pncA* gene n (%)	Crude Prevalence ratio (95%CI)	Adjusted Prevalence ratio (95%CI)	*P* value
Gender						
Male	211 (65.1)	136 (66.7)	75 (62.5)	1.0	1.0	
Female	113 (34.9)	68 (33.3)	45 (37.5)	1.12 (0.84–1.50)	11 (0.82–1.46)	0.56
Age (years)						
<40	153 (47.2)	87 (42.6)	66 (55.0)	1.0	1.0	
40–59	108 (33.3)	69 (33.8)	39 (32.5)	0.84 (0.61–1.14)	0.82 (0.60–1.11)	0.20
≧60	63 (19.4)	48 (23.5)	15 (12.5)	0.55 (0.34–0.89)	0.56 (0.34–0.91)	0.02
Residence in area within China						
East of china	104 (32.1)	69 (33.8)	35 (29.2)	1.0	NA	
Central of china	162 (50.0)	96 (47.1)	66 (55.0)	1.21 (0.87–1.68)	NA	
West of china	58 (17.9)	39 (19.1)	19 (15.8)	0.97 (0.61–1.54)	NA	
Occupationv						
Farmer	202 (62.3)	135 (66.2)	67 (55.8)	1.0	NA	
Others	122 (37.7)	69 (33.8)	53 (44.2)	1.31 (0.99–1.74)	NA	
Residence in area according to start DOTS implementation						
In 2000 or later	187 (57.7)	112 (54.9)	75 (62.5)	1.0	NA	
Before 2000	137 (42.3)	92 (45.1)	45 (37.5)	0.82 (0.61–1.10)	NA	
Number of previous treatments						
0	132 (40.7)	88 (43.1)	44 (36.7)	1.0		
1	107 (33.0)	67 (32.8)	40 (33.3)	1.12 (0.80–1.58)	1.17 (0.84–1.63)	0.35
More than 1	81 (25.0)	47 (23.0)	34 (28.3)	1.26 (0.89–1.79)	1.55 (0.90–1.84)	0.18
Unknown	4 (1.2)	2 (1.0)	2 (1.7)	1.50 (0.55–4.12)	2.56 (1.72–3.82)	< 0.001
Previous pyrazinamide usage						
Yes	144 (44.4)	93 (45.6)	51 (42.5)	1.0	NA	
No	180 (55.6)	111 (54.4)	69 (57.5)	1.08 (0.81–1.44)	NA	
Genotype						
Non-beijing strain	42 (13.0)	32 (15.7)	10 (8.3)	1.0	1.0	
Beijing strain	230 (71.0)	139 (68.1)	91 (75.8)	1.66 (0.95–2.92)	1.84 (1.01–3.34)	0.045
No data	52 (16.0)	33 (16.2)	19 (15.8)	1.54 (0.80–2.94)	1.64 (0.82–3.25)	0.16

Note: *NA* not applicable, these variables were not included in the final mode

### Association between drug susceptibility to pyrazinamide, and to ofloxacin and kanamycin

Table 4 shows a positive association between drug susceptibility for pyrazinamide versus ofloxacin and kanamycin. Pyrazinamide resistance proportions among ofloxacin-resistant and -susceptible isolates were 62.2 and 33.5% (*p* < 0.001), respectively. These proportions were 54.8 and 38.7% among kanamycin resistant and susceptible MDR-TB patients (*p* = 0.05), respectively.

**Table 4:** Association of phenotypic susceptibility to pyrazinamide, and to ofloxacin and kanamycin

Pyrazinamide susceptibility results	Ofloxacin susceptibility results			Kanamycin susceptibility results		
	Susceptible *n* (%)	Resistant *n* (%)	Total	Susceptible *n* (%)	Resistant *n* (%)	Total
Susceptible	161 (66.5)	31 (37.8)	192	173 (61.3)	19 (45.2)	192
Resistant	81 (33.5)	51 (62.2)	132	109 (38.7)	23 (54.8)	132
Total	242	82	324	282	42	324

## Discussion

Before a new RR/MDR treatment regimen composed of bedaquiline and linezolid can be implemented in the whole of China, pyrazinamide is still being considered an important optional drug in the treatment regimen of RR/MDR-TB given that such a regimen is widely accessible and currently more affordable. The hitherto available data from China showed a large variation in pyrazinamide prevalence as representative data were lacking. This is the first study assessing pyrazinamide resistance among MDR-TB cases from a nationwide representative sample. We showed that 40.7% of MDR-TB isolates exhibited resistance against pyrazinamide and 73.5% of these resistant isolates also showed genotypic resistance. Pyrazinamide resistance proportions among ofloxacin-resistant and kanamycin-resistant strains were both significantly higher than those among susceptible isolates. The reported prevalence of pyrazinamide resistance in MDR-TB isolates elsewhere varied substantially by country, ranging from 36.7 to 84.6% [17–21]. The range of previously reported proportions of pyrazinamide resistance in selected samples from China was 43.1–62.4% [11–14].

In our study, there were no obvious clinical characteristics significantly associated with phenotypic pyrazinamide resistance except for elderly MDR-TB patients less often having pyrazinamide resistance, as observed before in the USA [20]. One possible explanation is that the younger patients were recently infected with pyrazinamide resistant strains, while the elderly patients had reactivation of more remote infection, when pyrazinamide resistance levels were lower. Inadequate treatment, improper drug regimens, interrupted availability of drugs, or treatment for a very short period and an increased incidence of previous pyrazinamide exposure are possible causes for pyrazinamide resistance [21–23]. In one study conducted in Estonia and Latvia, findings were different as for the association between previous pyrazinamide usage and pyrazinamide resistance. In Estonia, the rates of resistance to pyrazinamide showed no difference between new and previously treated cases, whereas in Latvia, pyrazinamide resistance was higher in new cases than in previously treated cases [24]. Our present study also showed that there was no difference between pyrazinamide resistance in new cases versus in the previously treated cases. Patient’s age and lung cavities were previously reported to be associated with pyrazinamide resistance in MDR-TB cases [22], however, the extent to which age affects pyrazinamide resistance among MDR-TB cases was smaller compared with the effects of cavities and treatment history. Unfortunately, we do not have chest X-ray information on cavitation. One mathematical modeling suggested that resistance to second line injectable drugs is the result, rather than the cause, of accumulated resistance to pyrazinamide [25]. In our study, we saw an association between ofloxacin and kanamycin resistance on the one hand and pyrazinamide resistance on the other hand, but almost no full resistance profile among pyrazinamide resistant strains. So pyrazinamide may be a condition for ofloxacin and kanamycin resistance, which supports the above mathematical modeling that resistance of second-line drugs is the result of pyrazinamide resistance. More specific studies need to be conducted to investigate this hypothesis.

The high diversity of *pncA* mutations in pyrazinamide resistant strains is in contrast to the spectrum of mutations conferring resistance to rifampin and isoniazid. That the *pncA* gene accumulates multiple and diverse mutations may imply that there is a large target for resistance or resistance can result from multiple different variants acting individually or in concert. However, one study showed that an *M. tuberculosis* strain harboring a deletion in *pncA* conferring pyrazinamide resistance was estimated to be endemic longer before the use of pyrazinamide for TB treatment [26]. This finding supports the idea that purifying selection on *pncA* is relatively weak, which would contribute to its exceedingly high diversity and broaden the adaptive paths to resistance [27].

Among 132 pyrazinamide-resistant MDR-TB isolates, mutations in 97 isolates were scattered along the whole *pncA* gene. Mutations of the gene coding for pyrazinamidase, *pncA* is the major mechanism of pyrazinamide resistance. Detection of *pncA* gene mutations may provide pyrazinamide susceptibility information. The concordance between *pncA* gene mutations and pyrazinamide susceptibility varies substantially among previous studies, ranging from lower than 50% [28, 29] to higher than 75% [8, 10–12, 30]. Our finding was similar to that of most other studies. Scorpio et al reported that the distribution of *pncA* mutations clustered at three regions 3–7, 61–85, 132–142 [7]. Miotto et al reported that the most frequently affected regions (representing more than 70% of mutated strains) were found at the promoter(− 13 to − 3) and at codons 6 to 15, 50 to70, 90 to 100, 130 to 145, and 170 to 175 [31]. In our study, we found a high variety of *pncA* gene mutations in the MDR-TB isolates. Our findings support similar observations of widely dispersed mutations in previous studies. Although the basis of this diversity is unclear, it is hypothesized that either the gene is situated in a hot spot region, or that it is a consequence of the lack of DNA mismatch repair mechanisms in MTB [32]. For the mutations in *pncA*, 40/61 point mutations conferring pyrazinamide resistance that we found have been reported previously [33–35], and to our knowledge, 21 novel mutations and all 11 frameshift changes (insertions or deletions) were first found in this study. There are also other unknown resistance mechanisms responsible for the resistance phenotype, such as mutations in *rpsA* gene, however, low frequency of *rpsA* gene mutations showed that *rpsA* gene mutation was not the main mechanism of pyrazinamide resistance [36, 37]. Zimic et al have demonstrated that the efflux rate of pyrazinamide’s active moiety, pyrazinoc acid, predicts pyrazinamide resistance with up to 93% sensitivity [38]. Pyrazinamide acid efflux accounted for 61% of the variability in pyrazinamide susceptibility [39]. One study based on more than 100,000 genomes showed that the sensitivity of genotypic prediction of pyrazinamide resistance based on mutations in and upstream of *pncA* gene was 91.3% [40]. Whole genome sequencing cannot be routinely conducted and afforded in most areas of China due to technical challenges of whole genome sequencing and analysis of data. There is only one commercial molecular based assay (Genoscholar PZA-TB II) for pyrazinamide resistance detection, developed by the Nipro Corporation (Osaka, Japan), a line-probe assay that includes probes covering the entire *pncA* coding region and 18 nucleotides upstream [41]. This line-probe assay is unavailable in China, however.

The strength of the present study is that the MDR-TB strains were isolated from a random sample of patients distributed almost around the whole China (30/31 provinces) and were not restricted to only one or a few counties or provinces or one hospital setting. There are also some limitations. Firstly, some of the MDR-TB strains could not be recovered successfully. Secondly, the sample size of the original survey was not set to study geographic, clinical and demographic variation in pyrazinamide resistance, so our analyses may have failed to detect true differences in this regard. Thirdly, only the *pncA* gene was sequenced and other mechanisms possibly responsible for the pyrazinamide resistance were not explored. Fourthly, the strains and data were collected 12 years ago. It is to be expected that the prevalence of resistance has increased, in line with the finding of higher prevalence among the younger group of patients, who on average have been infected more recently than older patients. The higher prevalence in more recent years from several sub-national studies in China [11–14] also support this expectation. Further studies of larger samples collected during more recent years and exploring the resistance by using of whole genome sequence may provide more accurate and deeper insights into the mechanism and associated factors of pyrazinamide resistance in China. Fortunately the strains from routine drug resistance surveillance in recent years has been collected and transferred to the Chinese National Tuberculosis Reference Laboratory and we are planning to assess the pyrazinamide resistance status and even the trend of change during these years in a subsequent study.

## Conclusion

Pyrazinamide resistance among MDR-TB patients is common, especially in patients aged < 40 years in China. Pyrazinamide therefore should not be used as priority option of Group 3 drugs for treatment of RR/MDR-TB in China while awaiting scale up of new drugs and regimens including bedaquiline and linezolid if the susceptibility of pyrazinamide is not confirmed. The only *pncA*-based molecular method has certain value for detection of pyrazinamide resistance but is not as sensitive as molecular methods for detection of rifampin resistance such as XpertMTB/RIF. Other gene mutations conferring to pyrazinamide resistance still need to be explored to increase the predictive ability of molecular methods for pyrazinamide resistance testing.

## Supplementary information

**Additional file 1: Table S1.** Database for Univariable and multivariable analysis of risk factors for phenotypic and genotypic pyrazinamide resistance.

## Data Availability

All data generated or analysed during this study are included in this published article [and its supplementary information files].

## Abbreviations

MDR-TB: Multidrug-resistant tuberculosis
PRs: Prevalence ratios
RR-TB: Rifampin-resistant tuberculosis
XDR-TB: Extensively drug-resistant tuberculosis
